# Neglected ethical issues in biobank management: Results from a U.S. study

**DOI:** 10.1186/2195-7819-9-1

**Published:** 2013-03-27

**Authors:** R Jean Cadigan, Dragana Lassiter, Kaaren Haldeman, Ian Conlon, Erik Reavely, Gail E Henderson

**Affiliations:** 1 Department of Social Medicine, University of North Carolina, 27599-7240 Chapel Hill, NC USA; 2 Center for Genomics and Society, University of North Carolina, Chapel Hill, USA; 3 Department of Anthropology, University of North Carolina, Chapel Hill, USA; 4 Research Triangle Institute, Research, Triangle Park, NC USA; 5 Department of Sociology, University of North Carolina, Chapel Hill, USA

## Abstract

The empirical literature on the ethical, legal, and social implications (ELSI) of biobanking has almost entirely relied on the perspectives of those outside of biobanks, such as the general public, researchers, and specimen contributors. Little attention has been paid to the perspectives and practices of those who operate biobanks. We conducted a study of U.S. biobanks consisting of six in-depth case studies and a large online survey (N = 456), which was developed from the case study results. The case studies included qualitative interviews with a total of 24 personnel. Both interview and survey questions focused on how biobanks operate, and what policies and practices govern their relationships with specimen contributors and the researchers who use the specimens. Analysis revealed unexpected ethical dilemmas embedded in those policies and practices that highlight a need for practical planning. In this paper, we review three issues seldom explored in the ELSI literature: 1. the discrepancy between biobankers’ hope that the bank will exist “permanently” and the fact that funding is limited; 2. the lack of planning for what will happen to the specimens if the bank closes; and 3. the concern that once collected, specimens may be underutilized. These dilemmas are missing from current public representations of biobanks, which instead focus on the intrinsic value in storing specimens as essential to the advancement of translational research. We argue that attention to these issues is important for biobanking, and that greater transparency of these policies and practices will contribute to promoting public trust in biobanks.

## Introduction

As biobanks have proliferated in number and size over the past decade or more (Hoeyer 2012), they, and the “biobank research system” (Wolf et al. 2012), are often heralded as critical to the advancement of translational research. Interest in the ethical, legal, and social issues (ELSI) of biobanking has correspondingly proliferated over the past decade as researchers suggest policies and practices that preserve respect for the individuals who contribute specimens to the biobank while promoting research (Fullerton et al. 2010; Kaye 2012; Mitchell et al. 2011; Mongoven and Solomon 2012; Trinidad et al. 2011). The ELSI literature on biobanking has traditionally focused on concerns such as informed consent, specimen ownership, specimen contributor privacy and identifiability, and return of research results (Kaye 2012; Bledsoe et al. 2012; Clayton 2005; Faria 2009; McGuire and Gibbs 2006). More recently, researchers interested in the ELSI of biobanking have also focused on the importance of establishing trustworthy governance with transparent practices (Trinidad et al. 2011; Dabrock et al. 2012; O'Doherty et al. 2011).

Empirical data on ELSI concerns in biobanking have been gathered from various stakeholders including genome researchers, IRBs, specimen contributors, and the general public (Cadigan and Davis 2009; Capron et al. 2009; Dressler et al. 2012; Kaufman et al. 2009; Brothers et al. 2011). While some researchers have conducted empirical studies of biobanks (Hirtzlin et al. 2003; Salvaterra et al. 2012; Stranger et al. 2008; Hoeyer 2006; Johnson et al. 2012; Kaye et al. 2012; Pawlikowski et al. 2010), they are almost entirely focused on biobanks outside the United States. We are aware of no prior studies that have gathered substantial empirical data from members of biobanks within the United States. Thus, the perspectives of biobankers in this country have yet to be adequately incorporated into discussions of the ELSI of biobanking.

This paper reports on findings from our larger empirical study that gathered interview and survey data from U.S. biobankers on how biobanks operate, including their relationships with the individuals who contribute specimens and the researchers who use them. Our larger study is particularly focused on the development and implementation of ELSI-relevant policies and practices within biobanks. In this paper, we present three issues that emerged from our interview and survey data that we argue warrant further discussion in the ELSI literature and in the burgeoning literature on biobanking policies and practices. Each represents a disjuncture between how biobanks are marketed to participants and the general public as vehicles to advance translational research and how biobanks actually operate.

## Methods

Our mixed-methods study began with exploratory case studies of six biobanks conducted in 2011. Using results from the case studies, we created and piloted an online survey. We then revised the survey instrument and administered it to 456 biobanks in spring 2012. This project was approved by the IRB of the University of North Carolina, Chapel Hill.

### Case studies of six biobanks

Each case study included collection of documents (e.g., consent forms; material transfer agreements) and interviews. The case study biobanks were selected based on how they acquire specimens – *de novo* (collecting from individuals who contribute specimens directly to the biobank) and repurposed (acquiring specimens left over from clinical care); and whether they were part of a network^a^ of biobanks. We sampled two *de novo*, two repurposed, and two networked biobanks.

We interviewed individuals associated with each biobank case who filled the following member roles or oversight duties: founders or principal investigators, administrators or coordinators, bioinformatics specialists, internal ethics board representatives,^b^ and public relations specialists. Recognizing that not all biobanks would have individuals in these roles or titles, we created rough definitions of the duties. For each case, we recruited the biobank founder or principal investigator first and then consulted with him or her to identify potential respondents who performed the duties of interest. While almost all cases had individuals who filled the first three roles, not all had individuals who filled the latter two. For networked biobanks, we interviewed representatives from at least two sites within the network.

We interviewed 24 people across the six cases, using separate interview guides for each of the roles described above. While many of the questions appeared in more than one interview guide, some questions were role-specific. Interview topics included the history and mission of the biobank, its structure, policies and practices regarding certain ELSI considerations, the biobank’s relationships with the people who contribute specimens and the researchers who use them, and interviewees’ perceptions of the biobank’s successes and challenges. Interviews averaged 60 minutes, were digitally audio-recorded, and transcribed verbatim.

Four authors developed a codebook and used NVivo 9.0™, a qualitative data analysis software program, to code the data. Concordance of the coding was assessed repeatedly and inconsistencies were resolved through consensus. To analyze the data, the research team first read and discussed each transcript. Next, we generated reports for the most frequently utilized codes and summarized each code’s findings. Last, we developed an overview of each biobank that included summaries of ELSI-relevant topics, such as policies and practices related to informed consent, ensuring contributors’ confidentiality and privacy, ownership of specimens and data, and intellectual property rights. These biobank overviews also included summaries of the topics presented in the Results section below.

### Survey of biobanks

To conduct our survey, we created a list of biobanks in the U.S. by carrying out an exhaustive search for biobanks using PubMed, NIH RePORTER, and Google (Boyer et al. 2012). We defined a biobank as an “organization that acquires and stores human specimens and associated data for future research use.” The search process resulted in a list of nearly 800 biobanks, the majority based in academic organizations, with non-profit, governmental, and commercial also represented.

Prior to the main survey, we conducted a pilot survey to refine our measurements, with questions developed by consulting the case study results, prior ELSI research, and organizational sociology literature. Representatives from 100 biobanks from our list were recruited to complete the online survey. Data and comments provided in the pilot were used to modify the instrument for the main survey.

#### Sample

We primarily recruited the biobank contact person listed on the biobank’s website. We emailed a link to the online survey to contacts for the remaining 682 banks on our list. The contact person was almost always someone in an executive or managerial role within the biobank. While we recruited that contact person, s/he may have asked another member of the biobank to complete the survey. To be eligible to complete the survey, we asked that respondents have thorough knowledge of the policies and practices of the biobank. We also encouraged respondents to ask others in the biobank for information relevant to the survey, if necessary. Finally, we recommended that if someone still did not know a response to a question, to skip it. We concluded data collection in May 2012 with 456 completed interviews, a response rate of 72%.^c^ We gathered data on the title or role of the person completing the survey. Sixty-eight percent of respondents were in an executive role (for example, the PI, Director, CEO, or President), 29% were in a managerial role (such as coordinator, manager, or supervisor), and 3% filled some other role (such as a laboratory director or assistant, clinical recruiter, or a genetic counselor). Details about the survey and descriptive results regarding how biobanks are organized are found in Henderson et al. (2013).

## Results

The ELSI considerations discussed in the literature on biobanking are often framed around transparency and preserving respect for specimen contributors. Three issues emerged from our case studies and survey analysis that are relevant to transparency and respect for contributors, yet are seldom discussed in the ELSI literature: 1) a mismatch between how long the biobank intends to be in existence and the length of time funding is secured; 2) lack of planning for what will happen to biobank resources should the biobank close; and 3) underutilization of the biobank’s specimens and data by researchers.

### Hope for perpetuity, despite funding constraints

The biobanks in our case studies and survey receive funding through a variety of mechanisms with the vast majority relying on external funding to maintain operations. A recurrent theme in our case study interviews was concern regarding funding, specifically that funding would cease entirely or that it would be insufficient to cover costs. Despite this, most interview respondents said their biobank had no defined endpoint. For example, when asked about the expected lifespan of the biobank and its collections, one respondent commented, “Right now we don’t have a sunset on it… It could go on in theory forever,” while another member of the same biobank jokingly replied, “Well, we’ve all decided that we’re only going to plan for the first hundred years, and after that it’s somebody else’s job.” Other respondents acknowledged they had never considered whether or when the biobank might close:

I don’t think that I’ve heard conversation number one about that. I think the assumption has been that it continues until … there’s no longer either the will to do it or the resources to do it or a need for it any more.

A member of a non-profit, disease-focused biobank said the bank had no expected lifespan “because our mission basically is to put ourselves out of business…. Our mission is to solve [the disease].” However, she then quickly conceded the biobank’s funding limitations:

You know in the non-profit world these days you basically have to raise the money to stay in business. So I would love to say that we have a five-year or ten-year plan for the biobank, but these days you pretty much live year to year.

Talk of funding limitations was frequent in the case studies. One respondent commented that the biobank’s funding came with “no guarantees,” and acknowledged that, “If we were completely to run out of money, we would have to figure out what to do with the samples.” Another remarked that her biobank had only two to three years of funding in the current budget. When asked what problems that presented, she replied, “A lot. A lot of problems if it turns out that there’s no funding later on. I mean pretty much if we don’t get funding to continue it, we’ll just have to stop.” Despite her acknowledgement that biobank operations would cease, when asked whether specimen storage would stop, she commented, “The [specimens] are such a good resource, they would never just throw all the [specimens] away. So they’ll always be stored somewhere, and they’ll always be used.” This sentiment was not uncommon among respondents: somewhere, somehow the specimens would continue to be stored and used because they are too valuable to be destroyed.

In response to these case study results, we asked survey respondents whether specimen contributors are informed how long their specimens will be stored by the biobank (Table 1). Fifty-three percent of respondents indicated that their biobank informed contributors regarding how long specimens would be stored. Of these, 79% said contributors were told specimens would be stored “permanently,” while 4% gave a specific number of years ranging from 10 to 60 (Table 2). But even among biobanks that promised specimen contributors an end-date for storage of specimens, the number of years of intended storage far outlasted the period of current funding for each biobank. Finally, an additional 16% of respondents checked “something else,” and offered an open-ended explanation. Examples of these responses included “indefinitely” and “until samples are depleted.” While there are reasons why investigators offer optimistic estimates of how long specimens will be stored, it does not appear that many consider it appropriate to discuss this in connection with how long their funding period is or, indeed, how the financing of biobanking is organized.

**Table 1:** **Are specimen contributors informed how long specimens will be stored? (n = 426)** ^**a**^

Yes	225
	(52.8%)
No	140
	(32.9%)
Not sure	61
	(14.3%)

^a^ Missing data deleted listwise.

**Table 2:** **How long are contributors told their specimens will be stored? (n = 224)** ^**a**^

Permanently	178
	(79.5%)
Specific number of years	10
	(4.5%)
Something else ^b^	36
	(16.0%)

a Missing data deleted listwise.

^b^ Specific examples: “indefinitely;” “mixed model among contributing investigators;” “generally permanently, but the answer is specific to each protocol;” “indefinitely or until the biobank closes;” “until samples are depleted”.

When asked how concerned they were about running out of funding, 71% of respondents indicated that it is a “moderate” or “major” concern (30.6% moderate and 40.5% major) (Table 3). Moreover, in response to the open-ended question, “What would you consider to be the biobank’s greatest challenge?,” the most common response was funding (37%).

**Table 3:** **How much of a concern is funding? (n = 444)** ^**a**^

A major concern	180
	(40.5%)
A moderate concern	136
	(30.6%)
A minor concern	88
	(19.8%)
Not at all a concern	40
	(9.0%)

^a^ Missing data deleted listwise.

### Planning for closure of the biobank

Despite uncertain funding, our case study and survey data reveal that most biobanks do not have a plan for what to do with resources should the biobank close. Some respondents report never contemplating the idea, mostly because they have never considered that the biobank might end. For example, a member of a university medical center biobank stated:

…We don’t have a specific business continuity plan for if the resource were closed because we’re still in this phase of expanding the scope of the resource and realizing it was a better idea than we thought it was at the beginning…. So we’re still in the growth phase and still in the kind of optimistic “This is a way cool thing” kind of stage. And so I don’t think there’s been any notion that it would come to an end, but I suppose we could have some kind of disaster plan where we would decide to – I don’t know – destroy all the DNA samples.

While acknowledging that the bank could develop a “disaster plan,” this respondent indicates that a plan is unnecessary because the bank’s growth has exceeded original expectations. Like respondents from other biobanks, he notes that the bank lacks a contingency plan for dissolution should funding be discontinued because of its organizers’ focus on growth. As another respondent (from a network of biobanks) commented, “Plan A is to get funded and Plan B is to make Plan A work.” When asked to consider what would happen to the specimens and data should the network of biobanks fail to secure funding, he responded:

Let’s say we didn’t get re-funded if it were to happen. …The data repository would sort of just freeze in time because it wouldn’t have the funding to have future data downloads and that type of thing, and the specimens ultimately would be left at the [individual network] sites. And I suspect over time that … ownership would revert back to the site; you know, there it’s like possession is nine tenths of the law kind of thing. But I didn’t let myself go there because I don’t like to think about Plan B.

Because the network has no plan for the specimens in the absence of funding, this respondent guesses that they would be abandoned at the individual sites, and their ownership—another ELSI concern in biobanking—would be re-negotiated.

Our survey data reveal similar findings regarding the lack of a plan for specimens upon termination. We asked whether the biobank has “a written plan for what will happen to the specimens should the biobank be terminated for any reason.” Only 26% replied “yes” (Table 4). For those banks with a plan, we asked: “According to the plan, what will happen to the specimens should the biobank be terminated for any reason?” These open-ended responses included destruction, anonymization, use for other research, and transfer of the specimens to another biobank.

**Table 4:** **Does biobank have plan in case of termination? (n = 450)** ^**a**^

Yes	119
	(26.4%)
No	226
	(50.2%)
Not sure	105
	(23.3%)

^a^ Missing data deleted listwise.

The 74% of respondents who said the bank had no plan or were unsure, were asked, “In your opinion, what will happen to the specimens should the biobank be terminated for any reason?” While many offered responses similar to those with a specific plan, others balked at the thought of termination: “I don’t see it EVER being terminated. We have never contemplated this.” Another respondent believed, “Something would be worked out” by the university that houses the biobank.

### Underutilization of the specimens and data

Among the case study biobanks, large numbers of specimens and associated data are collected; however, many respondents expressed concern that the collections are underutilized by researchers. As one respondent lamented, “It’s totally false, and this was an eye-opener for me, that ‘if you build it they will come.’ I thought if we build this [biobank] we’ll have people knocking on our door to use it.”

Virtually all the case study biobanks struggle with trying to increase utilization of their specimens and data. For example, when asked how many requests for specimens and/or data they receive from researchers, a member of a biobank that features a collection of 1.6 million specimens taken from 1.3 million people said:

You know when you look historically the number has significantly increased, but it’s still relatively few. So if you look between 2000…and 2009…we would have one to two [requests] at most per year. In 2010 we had about six, and we’ve gotten two so far in 2011. So certainly, you know, a lot more than what we’ve had in the past, but still I think in this grand scheme of things, not a significantly large number.

A member of another biobank echoed this view:

Right now the concern I have is in the opposite direction. That we’re going to collect them, and they won’t be used. So right now I’m not too concerned about overuse, but I’d love to have that problem to be honest.

In fact, he placed concern with specimen utilization at the forefront of his work:

The question always is what wakes you up at 3:00 in the morning. The thing that wakes me up in regard to this project mostly is related to collecting a lot of specimens that sit in freezers…which is why I want to make sure they get used because the biggest criticism we got when we [applied for more funding] was “Yeah. We love this concept, but gosh, every time NIH [National Institutes of Health] has invested in a specimen repository, the specimens are collected and they aren’t used.” And I don’t want to see that happen.

In our survey, 69% of respondents said they were concerned that the specimens are underutilized (Table 5). We also asked how many requests the biobank receives each year for specimens and/or data (Table 6), revealing that 30% of the biobanks received five or fewer specimen requests per year. While the number of requests for specimens and/or data is not an exact measure of utilization in that it does not reveal the percentage of the specimens and/or data being used relative to the entire collection, it does, however, give an indication of the biobank’s success at marketing itself to researchers. In fact, following “funding,” the second most frequent response to the biobank’s greatest challenge was marketing the biobank to researchers (17%), which included concern about underutilization.

**Table 5:** **How much of a concern is underutilization of specimens? (n = 439)** ^**a**^

A major concern	57
	(13.0%)
A moderate concern	123
	(28.0%)
A minor concern	123
	(28.0%)
Not at all a concern	136
	(31.0%)

^a^ Missing data deleted listwise.

**Table 6:** **How many requests for specimens or data each year? (n = 417)** ^**a**^

0 to 5	125
	(30.0%)
6 to 15	93
	(22.3%)
16 to 50	89
	(21.3%)
51 to 100	50
	(12.0%)
More than 100	60
	(14.4%)

^a^ Missing data deleted listwise.

The struggle to get researchers to use specimens is in stark contrast to the missions articulated by the biobanks in our case studies, such as finding cures or treatments for diseases. This contrast was expressed by one interviewee who described her internal struggle over telling the people she recruits that the bank aims to revolutionize medicine, while knowing that the collection is not highly utilized:

I think we’ll be judged long-term by discoveries that are made using the samples that we’ve collected… I think you know this community [of participants] really, really wants to participate in something that has the chance to revolutionize medicine, and I’d really like to deliver on that.

## Discussion

This study examined biobanks’ policies and practices from the perspectives of those who conduct their work, giving us an opportunity to incorporate insiders’ experiences into our understandings of the ELSI of biobanking. Such bottom-up approaches (Meslin 2010) to examining policies and practices are vital to advancing the goal of promoting transparency and trust in biobanking. The three findings presented here—hope for perpetuity of the biobank despite funding constraints, lack of plan for the specimens should the biobank close, and perceived underutilization of the specimens and associated data—emerged during the course of the interviews and were corroborated by data from our larger survey. Others have argued that heightened expectations surrounding biobanks and other biotechnologies may cause people to set their hopes too high, while ignoring significant barriers, for what these technologies can offer future research (Petersen 2009). We are not in the position to evaluate the intrinsic value of collections, and it is certainly true that many factors will impact their ultimate success, including careful planning to meet an identified need and appropriate marketing to ensure utilization for that need. However, we contend that the discrepancy between biobankers’ optimism for results and the questionable conditions of sustainability, planning for termination, and specimen utilization, may inadvertently undermine transparency, informed consent, and ultimately, public trust.

The U.S. National Cancer Institute has noted the funding insecurities inherent in biobanking, and their recent efforts to discern the high financial costs of biobanking aim to highlight the importance of developing sustainable business models (Vaught et al. 2011). Our data on the mismatch between expectations for long term storage and the reality of short term funding confirm this need. Scientific research protocols require justifications based upon short and long term objectives, often memorialized in the required format for NIH proposals; and most funding is requested to accomplish those short term goals. In the case of biobanking, however, the justification for collection and storage shifts to long term, anticipated future uses of specimens and associated data, thus bringing into focus the conundrum of the mismatch.

While our case study data suggest that uncertain funding leads to deep concern about how to ensure continued financial support, it does not seem to lead to creating a strategic plan in the event that funding is lost. Our survey data verify this observation; we found no relationship between a biobank's level of concern about funding and whether the biobank has a plan for closure.^d^ terminated, often due to lack of funding. Moreover, best practice guidelines for biobanks recommend creating plans for closure (National Cancer Institute However, we know from recruiting survey respondents that biobanks *are*^2011^; International Society for Biological and Environmental Repositories 2012), even suggesting that the time to do so is at the earliest stages of the biobank’s creation (International Society for Biological and Environmental Repositories 2012, p.144).

Others have noted that closure of biobanks elicits important ethical and legal issues (Zawati et al. 2011; Janger 2005). Options after closure include destroying the specimens, transferring them to another facility, or letting them sit unused in freezers. Absent of destroying all specimens and data—actions that are of great concern to many biobankers and researchers (Cadigan et al. 2011a)—a number of questions arise. For example, should biobanks discuss possible closure with participants during the consent process, and if so, what should be the content of such discussions? If participants are not told during the consent process of a plan for closure, should they be informed upon termination of the biobank that their specimens and data will be transferred to a new facility, if that is the case? Should they be given the option of having them destroyed instead? If it is not possible to re-contact specimen contributors, who should decide the fate of the specimens and data, and based on what criteria? These issues and others we present need further discussion among those in the biobanking and ELSI communities, with careful attention paid to what is appropriate for different kinds of biobanks and different communities of contributors (Hoeyer 2010).

Biobanks operate on the promise of research that will be undertaken with the specimens. Indeed, that is their reason for being. Studies with people who have joined biobanks or genetic research studies reveal that many participate because they trust the biobank or researchers (Cadigan and Davis 2009; Cadigan et al. 2011b) and hope their participation will advance research that will ultimately help people (Cadigan et al. 2011b; Michie et al. 2011; Streicher et al. 2011). Biobanks’ own mission statements frequently include the broader goals of developing treatments or cures for disease. However, to support public trust in biobanks, it is important that the promise of biobanking and the research it facilitates not be overstated to participants. Moreover, as Zawati and colleagues (Zawati et al. 2011, p.431) remark, “Stemming from the emerging trend of reciprocity, striving to realize the full benefit of research to which participants have given their data and samples is considered by some as an ethical imperative, provided that confidentiality is protected.” If research is not being done, or is not being done in quantities that make significant advancement of science feasible, then is the promise broken and the public’s trust compromised?

The ELSI literature on biobanking has largely focused on the issues associated with a biobank’s relationship to its contributors, considering obligations represented in informed consent agreements and the potential obligation to return individual research results. While the biobank-contributor relationship is thoroughly explored in the ELSI literature, the equally important relationship between biobank and researcher remains woefully unexplored. Until now, biobank members’ concerns regarding how to attract researchers to use the specimens and data have never been examined. Moreover, the issue of researcher access and sharing, while addressed in the ELSI literature, has emphasized the importance of facilitating access rather than the problem of underutilization.

The three findings we present relate to developing and maintaining biobanks as trustworthy organizations. We argue that biobanks should be carefully planned and designed to meet an identified scientific need, and that these plans should include sustainable business models, appropriate marketing to ensure utilization, and plans for the specimens and data if the biobank is terminated. While optimism for biobanks’ role in translational research can be admirable, it must be accompanied by equal attention to building trusting relationships between biobanks and the public. Best practice guidelines suggest that trust is enhanced when a biobank’s policies and practices are transparent (National Cancer Institute 2011; International Society for Biological and Environmental Repositories 2012). (Zawati et al. 2011, p.431] argue that, “For biobankers, the most valuable asset is the trust of their participants in their endeavors, a trust that requires the provision of all necessary information during the consent process and the existence of reasonable and adequate mechanisms to protect their privacy and the security of their data and samples.”

The crux of the question is what constitutes “all necessary information,” and additionally, is the consent process the best way to convey it? For example, is it important for specimen contributors’ decision-making to know that the bank has limited funding? Would potential contributors join biobanking efforts knowing that if the biobank was forced to close there are no formal plans for their specimens and data? Is it important for participants to know rates of specimen and data use? How might that knowledge impact public trust and participation? Finally, how could some of these concerns be addressed? These questions are best answered through dialog with both specimen contributors and biobank researchers, as solutions will vary depending upon the particular biobank context. We hope that the questions raised by our research findings can be used to facilitate discussion about how biobank policies and practices may impact transparency and trust.

Our results also raise additional issues, well beyond the scope of this paper. These include the question of how underutilization is defined and best addressed, within the context of an individual bank’s scientific objectives. Perceived underutilization may or may not be linked to objective outcome measures. Our study also calls attention to the need to better understand forces in the broader environment that have facilitated the rise of the “biobank research enterprise” (Wolf et al. 2012) and consequences of the proliferation of biobanks, often in the face of scientific uncertainty.

## Endnote

^a^ We define a network as a group of biobanks that are formally linked and share resources; we call the individual biobanks within the network “sites.”

^b^ The individuals we interviewed who filled this role were not necessarily employed by the biobank, but had important roles in planning and developing the biobank and in oversight of its operations.

^c^ In the process of data collection, we discovered that 45 biobanks were ineligible. The total of biobanks presumed to be eligible was 637.

^d^ P = .82, data available upon request.
